# Variation in Research Experiences and Publications During Medical School by Sex and Race and Ethnicity

**DOI:** 10.1001/jamanetworkopen.2022.38520

**Published:** 2022-10-25

**Authors:** Mytien Nguyen, Sarwat I. Chaudhry, Emmanuella Asabor, Mayur M. Desai, Elle Lett, Jose E. Cavazos, Hyacinth R. C. Mason, Dowin Boatright

**Affiliations:** 1 MD-PhD Program, Yale School of Medicine, New Haven, Connecticut; 2 Section of General Internal Medicine, Department of Medicine, Yale School of Medicine, New Haven, Connecticut; 3 Department of Chronic Disease Epidemiology, Yale School of Public Health, New Haven, Connecticut; 4 Computational Health Informatics Program, Boston Children’s Hospital, Boston, Massachusetts; 5 Perelman School of Medicine, University of Pennsylvania, Philadelphia; 6 South Texas Medical Scientist Training Program, University of Texas Health San Antonio, San Antonio; 7 Tufts University School of Medicine, Boston, Massachusetts; 8 Department of Emergency Medicine, New York Grossman School of Medicine, New York

## Abstract

**Importance:**

Diverse research teams are critical to solving complex health problems and producing high-quality medical research.

**Objective:**

To examine the associations of student sex and racial and ethnic identity with publication rates during medical school.

**Design, Setting, and Participants:**

This cohort study assessed individual-level data of US MD graduates from medical school who matriculated in academic years 2014 to 2015 and 2015 to 2016. Data were obtained from the Association of American Medical Colleges and analyzed from October 2021 to January 2022.

**Main Outcomes and Measures:**

Outcomes of interest included students’ self-reported participation in unique research experiences, number of publications, and computed publications per research experience. Poisson regressions were constructed to determine the association of sex and racial and ethnic identity with research outcomes using adjusted rate ratios (aRRs).

**Results:**

Among 31 474 graduates, 15 159 (48.2%) identified as women and 4344 (13.8%) identified as underrepresented in medicine by race and ethnicity (URIM; including American Indian, Alaska Native, Black, Hawaiian Native, Hispanic/Latinx, and Pacific Islander individuals). Students who attended National Institutes of Health (NIH) top 40 research-ranked schools reported higher number of research experiences and publication counts, resulting in a higher publication rate compared with students from non–top 40 schools (median [IQR] 1.60 [1.00-3.00] vs 1.25 [0.50-2.33]; *P* < .001). Women reported a higher number of research experiences than men but a significantly lower number of publications (top 40 schools: aRR, 0.89; 95% CI, 0.87-0.90; non–top 40 schools: aRR, 0.93; 95% CI, 0.92-0.95). This resulted in a significantly lower publication rate among women (top 40 schools: aRR, 0.85; 95% CI, 0.83-0.86; non–top 40 schools: aRR, 0.91; 95% CI, 0.90-0.92). Compared with White students, Asian students had higher publication rates at both NIH top 40 schools (aRR, 1.10; 95% CI, 1.08-1.12) and non–top 40 schools (aRR, 1.07; 95% CI, 1.05-1.08), while lower publication rates were reported among Black students (top 40 schools: aRR, 0.83; 95% CI, 0.80-0.86; non–top 40 schools: aRR, 0.88; 95% CI, 0.85-0.95) and Hispanic students attending non–top 40 schools (aRR, 0.93; 95% CI, 0.90-0.95).

**Conclusions and Relevance:**

These findings illustrate that inequities in the physician-scientist workforce began early in training and highlight key areas for intervention, such as funding support and mentorship training during undergraduate medical education, that may promote the future success of a diverse physician-scientist workforce.

## Key Points

**Question:**

Are student sex and racial and ethnic identity associated with publication rate during medical school?

**Findings:**

This cohort study of 31 474 medical school graduates found a significant disparity in publication rates across sex and race and ethnicity, with women and Black and Hispanic students reporting lower publication rates compared with men and White students. Sex and racial and ethnic disparities in research persisted at both National Institutes of Health top 40 and non–top 40 research-intensive schools.

**Meaning:**

These findings suggest that inequities in the physician-scientist workforce began early in training, as exposure to research and research productivity are critical for a successful career as a physician-scientist.

## Teaser

This cohort study examines the associations of student sex and racial and ethnic identity with publication rates in medical school.

## Introduction

Diverse research teams have a greater ability to solve complex problems, produce higher quality research, and garner more participation from racial and ethnic groups typically underrepresented in clinical trials, such as including American Indian, Alaska Native, Black, Hawaiian Native, Hispanic/Latinx, and Pacific Islander individuals.^1,2,3,4^ Despite the need for a more diverse biomedical workforce, racial, ethnic, and sex inequities in representation among physician-scientists remain high. Women and underrepresented in medicine (URIM; including American Indian, Black, and Hispanic/Latinx) physician-scientists constitute only small proportions of National Institutes of Health (NIH)–funded physician-scientists (women: 25%; URIM individuals: 5%).^5,6^ Furthermore, compositional representation among women, URIM, and Asian faculty decreases with faculty rank and leadership.^7,8^ The NIH and National Academies of Sciences, Engineering, and Medicine have recommended addressing this cumulative attrition in representation as a critical intervention to increase diversity in the physician-scientist workforce.^5,9,10,11^

While interest in a career as a physician-scientist can develop during many stages in professional development, experiences during medical school play a critical role in shaping both interest and future success in research careers. However, few studies have examined the learning environment for research during medical school for URIM and women students. Greater understanding of the learning environment for URIM and women medical students pertaining to research is important because prior studies have shown that experiences of discrimination during medical school^12^ are associated with adverse outcomes for students historically marginalized in medicine, including higher rates of mistreatment, burnout, leave of absence, and attrition.^13,14,15,16,17,18,19,20^ The racialized experiences of marginalized students in the medical school learning environment could have a deleterious impact on the experiences of women and URIM students and ultimately the diversity of the biomedical research workforce.

To better understand the educational experiences of women and URIM students during these formative years, we examined the associations of sex and racial and ethnic identity, and the intersection of sex and racial and ethnic identities, with research outcomes among a national sample of recent medical school graduates. We explored several metrics for research outcomes, including number of research opportunities students participated in, total number of publications, and number of publications per research experience (ie, publication rate). We hypothesize that URIM and women students, especially students who identify with both marginalized identities, would have less exposure to research experiences and a lower publication rate compared with men and White students.

## Methods

This retrospective cohort study was deemed exempt from review and informed consent by the Yale School of Medicine institution review board because data were deidentified. This study is reported following the Strengthening the Reporting of Observational Studies in Epidemiology (STROBE) reporting guideline.

### Data and Participants

We conducted an analysis of all US medical school MD graduates who matriculated in the 2014 to 2015 and 2015 to 2016 academic years and were applying to residency from 2018 to 2021. Our study sample excluded students graduating from dual-degree programs, such as MD-PhD, MD-MBA, and MD-MPH, due to the variety of research experience among dual-degree students and that research is embedded into the curriculum of many dual-degree programs. We obtained deidentified student-level data from the Association of American Medical Colleges (AAMC) Electronic Residency Application Service (ERAS) and Student Records System.

### Medical Student Demographic Characteristics

Student characteristics included self-reported race and ethnicity and self-reported sex (woman or man). Due to data limitations, we were unable to include non-cisbinary gender identities. Students’ race and ethnicity were categorized as Hispanic (regardless of race), non-Hispanic American Indian or Alaska Native, non-Hispanic Asian, non-Hispanic Black, non-Hispanic Hawaiian Native or other Pacific Islander, or non-Hispanic White. Non-Hispanic individuals who reported more than 2 racial or ethnic identities were categorized as non-Hispanic multiracial. Our data did not include racial disaggregation of Hispanic ethnicity. Students who reported unknown or other race (3082 students [8.9%]) did not provide further race and ethnicity information and were excluded from our study cohort. URIM students included students who identified as American Indian or Alaska Native, Black, Hawaiian Native or other Pacific Islander, or Hispanic, alone or in combination with another racial or ethnic category.

### Research Experiences, Publications, and Productivity

Our primary outcome was students’ rate of publication per research experience during medical school. To determine rate of publication, we first examined the number of research experiences and publication count reported by students on their ERAS application. Publications included peer-review articles, book chapters, and published abstracts.

### Covariates

Covariates included Medical College Admissions Test (MCAT) scores, previously shown to be associated with number of publications,^21^ categorized into quartiles. Additionally, because some students elect to spend additional time in medical school to pursue research experiences, we accounted for the total time in years that a student spent in medical school, categorized into 2 levels: greater than 4 years or less than or equal to 4 years. Because the research intensity of each medical school may influence research opportunities afforded to students, we considered the ranking of medical schools from which students graduated from based on NIH funding (top 40 or not top 40), as provided by the AAMC.^22^

### Statistical Analysis

Our study cohort was stratified by NIH research funding status and examined differences in the mean number of research experiences, publications, and rate of publication using *t* test and 1-way analysis of variance (ANOVA) of the log-transformed counts with Tukey test for multiple comparisons where appropriate. We then used zero-inflated Poisson regression to model the association between self-reported sex and racial and ethnic identity, as well as intersections between sex and racial and ethnic identity, with publication rate. Multivariable analysis adjusted for demographic factors, MCAT score, and time to graduation. *P* values were 2-sided, and statistical significance was set at *P* < .05. Statistical analyses were performed using Stata statistical software version 16.1 (StataCorp). Data were analyzed from October 2021 to January 2022.

## Results

Our study sample included 31 474 MD graduates from medical school who matriculated in academic years 2014 to 2015 and 2015 to 2016; 15 159 graduates (48.2%) identified as women, 4344 graduates (13.8%) identified as URIM, and 8976 graduates (28.5%) graduated from medical schools ranked in the top 40 for NIH research funding (eTable 1 in the Supplement). Compared with women and URIM students, a higher proportion of White students and men graduate in 4 years or less and scored in the top MCAT quartile (eTable 2 in the Supplement). Overall, 28 848 students (91.6%) in our cohort reported 1 or more research experiences during medical school.

Student composition and research outcomes were significantly different between schools ranked in the top 40 and not top 40 in total NIH research funding. Compared with students in NIH top 40 research-funded medical schools, students graduating from medical schools not ranked in the top 40 were more likely to identify as women and Hispanic, and report fewer research opportunities and total publications (eTable 1 in the Supplement). Notably, students graduating from medical schools not in the top 40 by NIH funding reported significantly fewer publications per research experience than those who attended schools in the top 40 (median [IQR] publication rate, 1.25 [0.50-2.33] vs 1.60 [1.00-3.00]; *P* < .001) (eTable 1 in the Supplement).

### Research Experiences by Sex and Race and Ethnicity 

**Figure 1.**
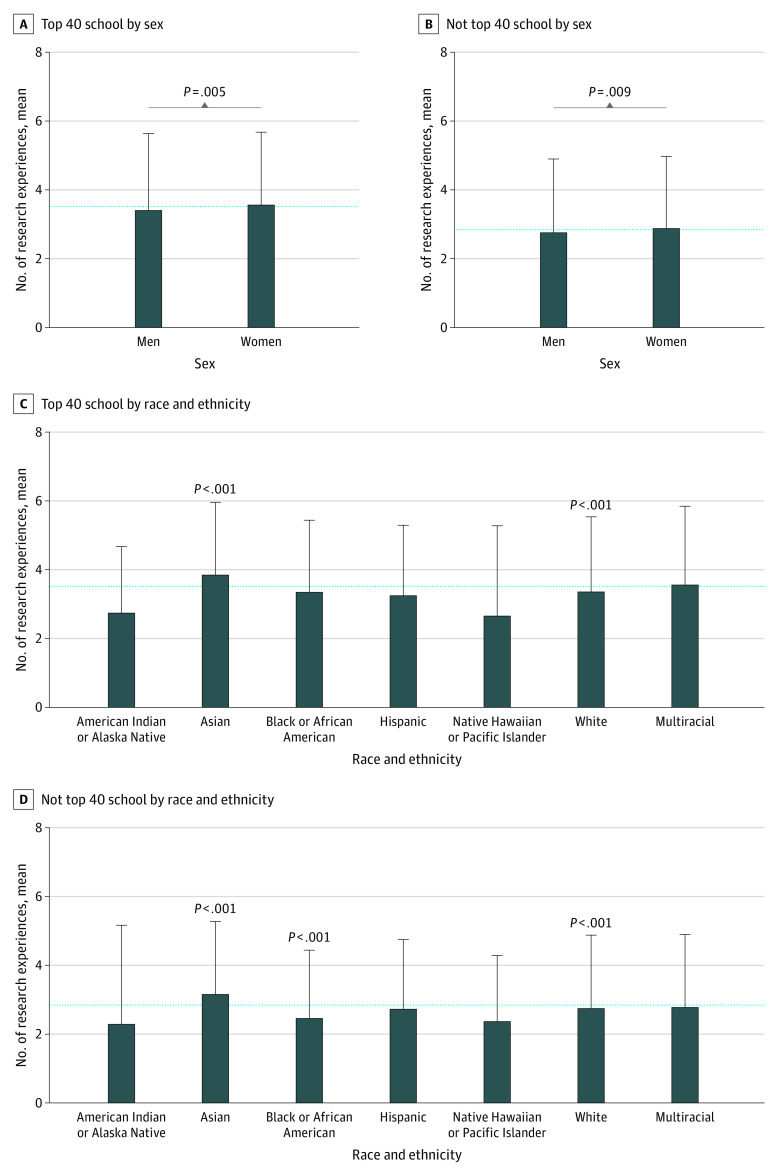
Total Number of Research Experiences Among MD Graduates From US Medical Schools by Sex and Race and Ethnicity Mean number of research experiences stratified by medical school National Institutes of Health research ranking. Significant analysis of variance test of log-transformed research experiences count with Tukey adjustment for multiple comparisons among groups are shown. Dashed line indicates the overall mean research experiences among all students; error bars, SDs.

At both NIH top 40 and non–top 40 medical schools, women had significantly more research experiences compared with men (Figure 1A and B). At non–top 40 schools, women had a mean (SD) of 2.90 (2.06) research experiences, while men reported a mean (SD) of 2.78 (2.11) research experiences (ANOVA *P* = .009). At top 40 medical schools, women reported a mean (SD) of 3.59 (2.08) research experiences and men reported a mean (SD) of 3.43 (2.20) research experiences (ANOVA *P* = .005).

Among all racial and ethnic groups, Asian students reported the highest number of research experiences (top 40 schools: mean [SD], 3.88 [2.09] experiences; non–top 40 schools: mean [SD], 3.19 [2.09] experiences) (Figure 1C and D). At NIH non–top 40 medical schools, while White students reported a mean (SD) of 2.78 (2.10) research experiences, Black students reported 2.49 (1.95) research experience (ANOVA *P* < .001) (Figure 1D). However, at top 40 medical schools, there was no significant difference in research opportunities between White and URIM students (Figure 1C).

### Total Publications by Sex and Race and Ethnicity

**Figure 2.**
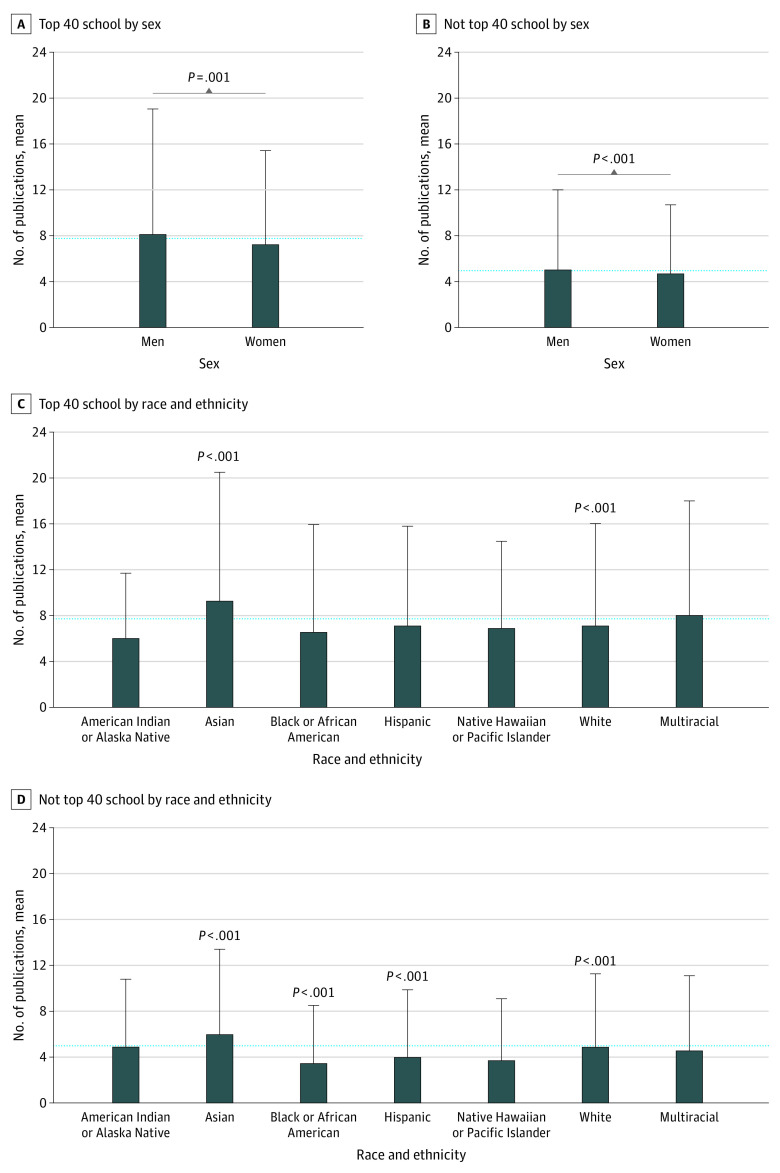
Publication Counts Among MD Graduates From US Medical Schools by Sex and Race and Ethnicity Number of publications (including peer-review articles, abstracts, presentations) stratified by medical school National Institutes of Health NIH research ranking (top 40 vs not top 40). Significant analysis of variance test of log-transformed research experiences count with Tukey adjustment for multiple comparisons among groups are shown. Dashed line indicates the overall mean publication count for all students; error bars, SDs.

In unadjusted analysis, women had fewer publications compared with men at both NIH top 40 (mean [SD], 7.32 [8.11] publications vs 8.22 [10.84] publications; *P* = .001) and non–top 40 (mean [SD], 4.81 [5.92] publications vs 5.15 [6.88] publications; *P* < .001) medical schools (Figure 2A and B). After adjusting for MCAT score and time to graduation, women still had fewer total publications compared with men at top 40 (aRR, 0.89; 95% CI, 0.87-0.90) and non–top 40 (aRR, 0.93, 95% CI, 0.92-0.95) medical schools (eTable 3 in the Supplement).

Among racial and ethnic groups, Asian students reported the highest mean number of publications, followed by White and multiracial students (Figure 2C and D), while URIM students reported the lowest mean number of publications (Figure 2C and D). In adjusted analyses, the total publication count was significantly higher among Asian students compared with White students at both NIH top 40 (aRR, 1.22; 95% CI, 1.20-1.24) and non–top 40 (aRR, 1.13; 95% CI, 1.12-1.15) medical schools (Figure 2; eTable 3 in the Supplement), while Black students (aRR, 0.87; 95% CI, 0.84-0.89) and Hispanic students (aRR, 0.96; 95% CI, 0.94-0.99) students had significantly fewer publications than their White peers at non–top 40 medical schools. At top 40 schools, while there was no significant difference in total publication count between Black and White students, Hispanic students reported more total publications compared with White students (aRR, 1.10; 95% CI, 1.06-1.14).

### Publication Rates by Sex and Race and Ethnicity

In adjusted analyses, women had significantly lower publication rates than men, with the largest sex difference at NIH top 40 medical schools (top 40 schools: aRR, 0.85; 95% CI, 0.83-0.86; non–top 40 schools: aRR, 0.91; 95% CI, 0.90-0.92) (Table). Similar inequities existed for URIM groups. In adjusted analyses, compared with White students, Asian students had a significantly higher publication rate at both NIH top 40 (aRR, 1.10; 95% CI, 1.08-1.12) and non–top 40 (aRR, 1.07; 95% CI, 1.05-1.08) medical schools, while Black students had significantly lower publication rates (top 40 schools: aRR, 0.83; 95% CI, 0.80-0.86; non–top 40 schools: aRR, 0.88; 95% CI, 0.85-0.95) (Table). Although Hispanic students had similar publication rates to White students at NIH top 40 medical schools, they had a lower publication rate at non–top 40 schools (aRR, 0.93; 95% CI, 0.90-0.95) (Table).

**Table.** Association of Sex and Race/Ethnicity With Publication Rate Among 2018-2020 MD Graduates

Group	Medical School NIH Research Ranking					
	Top 40			Not Top 40		
	Publication rate, median (IQR), No.	RR	aRR^a^	Publication rate, median (IQR), No.	RR	aRR^a^
Sex						
Men	1.67 (1.00-3.00)	1 [Reference]	1 [Reference]	1.33 (0.50-2.50)	1 [Reference]	1 [Reference]
Women	1.50 (1.00-2.71)	0.85 (0.84-0.87)	0.85 (0.83-0.86)	1.25 (0.50-2.25)	0.91 (0.89-0.92)	0.91 (0.90-0.92)
Race and ethnicity						
American Indian/Alaska Native	2.00 (1.00-3.25)	1.07 (0.92-1.25)	1.01 (0.87-1.18)	1.42 (0.00-2.50)	1.10 (0.95-1.26)	1.11 (0.97-1.28)
Asian	1.80 (1.00-3.00)	1.13 (1.11-1.15)	1.10 (1.08-1.12)	1.50 (0.75-2.50)	1.07 (1.05-1.08)	1.07 (1.05-1.08)
Black	1.33 (0.75-2.40)	0.94 (0.91-0.97)	0.83 (0.80-0.86)	1.00 (0.33-2.00)	0.84 (0.81-0.86)	0.88 (0.85-0.9)
Hawaiian Native or other Pacific Islander	1.96 (0.72-2.80)	1.20 (0.97-1.48)	1.08 (0.87-1.33)	1.25 (0.60-2.00)	0.99 (0.80-1.23)	1.00 (0.81-1.23)
Hispanic	1.50 (0.75-3.00)	1.08 (1.05-1.12)	1.00 (0.97-1.04)	1.00 (0.25-2.00)	0.90 (0.88-0.93)	0.93 (0.90-0.95)
White	1.60 (1.00-3.00)	1 [Reference]	1 [Reference]	1.33 (0.60-2.40)	1 [Reference]	1 [Reference]
Multiracial	1.50 (1.00-3.00)	1.04 (1.01-1.07)	0.99 (0.96-1.02)	1.00 (0.50-2.00)	0.97 (0.95-1.00)	0.99 (0.96-1.01)

Abbreviations: aRR, adjusted rate ratio; NIH, National Institutes of Health.

^a^ Adjusted for Medical College Admissions Test score and time to graduation.

### Interaction of Sex With Race and Ethnicity in Publication Rate

**Figure 3.**
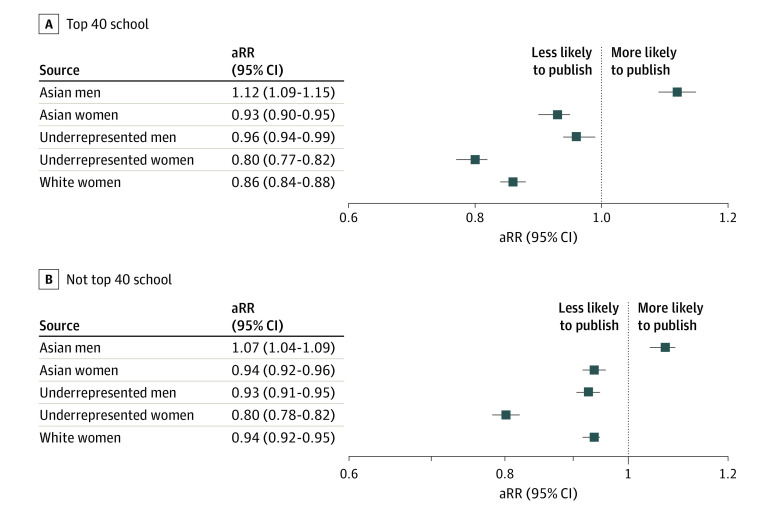
Intersections of Sex With Race and Ethnicity Among Medical Students Adjusted rate ratios (aRRs) of publication rate, publication counts per research experience, stratified by medical school National Institutes of Health research ranking compared with White men as the reference group, adjusted for Medical College Admissions Test score and time to graduation. Underrepresented in medicine includes students identifying as American Indian or Alaska Native, Black, Hispanic, Hawaiian Native or other Pacific Islander, and multiracial.

In our intersectional analysis by sex and racial and ethnic identity, URIM women had the lowest publication rate across all groups, which was at least 20% lower than White men at both top 40 (aRR, 0.78; 95% CI, 0.72-0.82) and non–top 40 (aRR, 0.75; 95% CI, 0.71-0.78) schools (Figure 3). Notably, there were significant sex differences in publication rate irrespective of racial and ethnic identity (Figure 3). While Asian students overall had high publication rates (Table), Asian women had approximately 6% lower rate of publication compared with White men at both NIH top 40 (aRR, 0.93; 95% CI, 0.90-0.95) and non–top 40 (aRR, 0.94; 95% CI, 0.92-0.96) medical schools (Figure 3). URIM women and men had significantly lower publication rates compared with White men at both NIH top 40 and non–top 40 schools (Figure 3).

## Discussion

In this cohort study, we identified significant patterns of racial and ethnic and sex inequity in publication rates during medical school, a formative period in the professional identity of physician-scientists. Women and URIM students had disparate research outcomes compared with men and White students. Women had a mean of 10% fewer publications, while Black students had approximately 15% fewer publications, and Hispanic students had approximately 7% fewer publications. Even within NIH top 40 schools, where research opportunities were more equitable, women and URIM students reported significantly fewer publications per research experience. Notably, URIM women had the lowest publication rate across all groups, with a gap of 20% between URIM women and White men.

Remarkably, we found significant parallels in sex and racial and ethnic inequities among medical graduates and the current physician-scientist workforce. The inequity for Black medical students, specifically Black women students, reflect current inequity that Black and women faculty encounter, including lower likelihood of NIH grant application and funding success.^23^ This suggests that investment in understanding effective interventions along the path to an independent research career at both the national and institutional level could have far-reaching implications for the biomedical workforce.

While inequities in research exposure and authorship opportunities during medical school may be due to a multitude of factors, women and URIM medical students have often reported a lack of mentorship opportunities compared with their peers.^24^ Men and non-URIM students are more likely to have access to and work with high-impact mentors with demonstrated research productivity and extensive research networks^24^ and thus are more likely to acquire the necessary tacit knowledge and social capital that facilitates future success in securing research funding and career advancement.^25^

Furthermore, due to negative stereotypes, mentors may withhold support from URIM mentees until they have a record of success, ie, proving themselves worthy of the support.^25^ Mentorship training that highlights how race- and sex-stereotype biases impact the mentor-mentee relationship and mentee success could be a promising intervention improve current mentorship models to promote the advancement of women and URIM students.

Our study found lower publication rates for women and URIM students despite exposure to a similar number of research opportunities. While this finding warrants further investigation, prior studies have shown that the scientific work and ideas of women and URIM investigators are often not valued as highly as those of their counterparts.^4^ This devaluation of academic products has also been demonstrated in medical education, as women are 59% less likely to receive research awards compared with men and URIM students are 82% less likely to receive research awards compared with non-URIM students.^26^

While we found that Asian students reported a higher publication rate compared with White students, our intersectional analysis revealed that this observation was driven by Asian men. Despite the high publication rate of Asian men, it is important to note that Asian men continue to experience significant inequity in medical training, including racial discrimination,^18^ lower rates of membership in honor societies,^13,27,28^ higher rates of attrition from medical school,^15^ and lower placement rates into graduate medical education training.^29^

Unlike Asian men, Asian women had significantly lower publication rates compared with White men at both NIH top 40 and non–top 40 schools. This disparity could have implications for the known sex disparities in the biomedical research workforce and highlights the critical lens of intersectionality in diversity and inclusion. Although Asian representation in medicine declines with academic rank, the disparity in representation is largest for Asian women faculty in leadership positions.^30,31^

Notably, the magnitude of inequities in research exposure and opportunities for authorship was greater in medical schools that were not ranked in the top 40 by NIH research funding. Non–top 40 medical schools may have an additional social mission to train physicians that serve the local communities, thereby selecting a group of medical students who may be less focused on research. Competing and different priorities between these schools and the NIH may lead to relatively smaller NIH funding to these schools.

Although we may anticipate fewer overall publications among medical students at medical schools not ranked in the top 40 by NIH funding compared with more research-intensive institutions, the disparate outcomes in publication count and rate among medical students by sex and racial and ethnic identity at medical schools not ranked in the top 40 by NIH funding is conspicuous. It is possible that there are fewer opportunities for research at schools ranked outside of the NIH top 40, creating a sense of scarcity in these institutions, and prior literature has demonstrated that discrimination is more prevalent when there is economic scarcity.^32^

These findings illustrate the potential role of NIH support in mitigating sex and racial and ethnic disparities in research outcomes among trainees. Although NIH resources have been concentrated among top 40 medical schools, NIH advisory committees have called for a distribution of support to schools that currently lack significant NIH support.^5^ Increasing NIH funding to students at non–top 40 schools in the form of training grants, diversity supplements, and individual fellowships may reduce the profound sex and racial and ethnic disparity found in non–top 40 schools.

### Implications

Our study findings have implications for national funding agencies and medical school governing bodies. The NIH, the largest funder of biomedical research, has made considerable efforts in recent years to improve diversity, equity, and inclusion at all levels, including the UNITE initiative.^33,34^ Programs that reward mentorship of URIM students should also be expanded to enhance impact. Such programs include diversity supplements^35^ and the NIH’s Administrative Supplements to Recognize Excellence in Diversity, Equity, Inclusion, and Accessibility Mentorship.

In addition, our findings may have implications for the Liaison Committee on Medical Education (LCME), the accrediting body for all MD-granting institutions. Students reporting productive research experiences and publications while in medical school are significantly more likely to pursue scientific careers and obtain NIH funding as faculty.^21,36^ While the LCME currently requires all MD-granting institutions to provide scholarly and research opportunities for students as an accreditation standard, the LCME currently does not examine aspects of equity and inclusion in these opportunities at the medical school level. Differences in research experiences, publications, and publication rates may represent important measures of equity that could be assessed by the LCME during future accreditation process. These measures would be consistent with the LCME’s current diversity accreditation standard and would also represent an evidence-based measure to bolster the future diversity of the biomedical research enterprise.

### Limitations

Our study has several limitations. This is a retrospective cohort study and represents observational data on disparity in research outcomes. Future qualitative studies designed to test the causal mechanisms of sex and racial and ethnic inequity, including when this inequity arises, are critical to provide more informed recommendations for transformational intervention. Publications and research experiences were self-reported and are dependent on individuals’ interpretation of what constitutes a unique research experience. Self-reported publications reported by graduates on the ERAS included research articles, peer-review abstracts, and book chapters. Although not all publications are original research articles, abstracts and book chapters are also important research activities that indicate a students’ contribution to the project and ability to communicate about the research. Additionally, although the sample sizes for American Indian or Alaska Native and Hawaiian Native or other Pacific Islander students were small, the CIs were reasonably narrow. American Indian or Alaska Native and Hawaiian Native or other Pacific Islander student populations have traditionally been invisible in medical education research, often grouped as others, due to significant inequity in recruitment and retention. Because the small sample size reflects existing inequity and not a problem with sampling, we opted to report statistics for these groups independently. Similarly, Asian students are a heterogenous group of students with significantly varied histories of marginalization and immigration in the US. Although we do not have disaggregated Asian subgroup data for this study, it is critical to examine research outcomes of Asian students across subgroups in future research. Additionally, prior research has indicated the importance of mentor characteristics, such as training history, tenure status, and seniority, on students’ productivity. Students’ motivation to pursue research and publications may also vary across specialty. Although our study did not include students’ specialty choice or mentor characteristics, future research should evaluate the influence of specialty choice and mentor characteristics on students’ research exposure and opportunities for authorship.

## Conclusions

This cohort study demonstrated significant inequity in research exposure and outcome for female and URIM students. Research exposure and productivity are critical for a successful career as a physician-scientist. Prior research has demonstrated the importance of research experiences during medical school, which are significantly associated with future full-time faculty appointment^21^ and receipt of mentored K awards among US medical school graduates.^37^ Mitigating differences in research exposure and authorship opportunities at the medical student level may help alleviate racial and ethnic and sex inequity in NIH funding among faculty and increase diversity in the physician-scientist workforce.

## Notes

Supplement. **eTable 1.** Characteristics of US MD Graduates Between 2018 to 2019 and 2019 to 2020 by Medical School NIH Research Funding Rank **eTable 2.** Characteristics of US MD Graduates Between 2018 to 2019 and 2019 to 2020 by MCAT Score and Time to Graduation **eTable 3.** Association of Sex and Race and Ethnicity With Publication Count Among 2018 to 2020 MD Graduates
