# Supplementary material for: Variation in Research Experiences and Publications During Medical School by Sex and Race and Ethnicity
Source: JAMA Netw Open. 2022 Oct 25;5(10):e2238520. doi: 10.1001/jamanetworkopen.2022.38520 (PMC9597391; doi:10.1001/jamanetworkopen.2022.38520)
Supplement: Supplement. — eTable 1. Characteristics of US MD Graduates Between 2018 to 2019 and 2019 to 2020 by Medical School NIH Research Funding Rank eTable 2. Characteristics of US MD Graduates Between 2018 to 2019 and 2019 to 2020 by MCAT Score and Time to Graduation eTable 3. Association of Sex and Race and Ethnicity With Publication Count Among 2018 to 2020 MD Graduates [file jamanetwopen-e2238520-s001.pdf]

Supplemental Online Content

Nguyen M, Chaudhry SI, Asabor E, et al. Variation in research experiences and publications during medical school by sex and race and ethnicity. *JAMA Netw Open*. 2022;5(10):e2238520. doi:10.1001/jamanetworkopen.2022.38520

eTable 1. Characteristics of US MD Graduates Between 2018 to 2019 and 2019 to 2020 by Medical School NIH Research Funding Rank
eTable 2. Characteristics of US MD Graduates Between 2018 to 2019 and 2019 to 2020 by MCAT Score and Time to Graduation
eTable 3. Association of Sex and Race and Ethnicity With Publication Count Among 2018 to 2020 MD Graduates

This supplemental material has been provided by the authors to give readers additional information about their work.

© 2022 Nguyen M et al. *JAMA Network Open.*

eTable 1. Characteristics of US MD Graduates Between 2018 to 2019 and 2019 to 2020 by Medical School NIH Research Funding Rank

	**Medical School NIH Research Ranking**		***p*-value**
	**Top 40**	**Not Top 40**	
**Total: 31,474**	N=8,976	N=22,498	
**Sex**			<0.001
Male	4,617 (51.4%)	10,542 (46.9%)	
Female	4,359 (48.6%)	11,956 (53.1%)	
**Race/ethnicity**			<0.001
NH White	5,150 (57.4%)	13,483 (59.9%)	
NH Asian	2,133 (23.8%)	4,296 (19.1%)	
NH Black/African American	542 (6.0%)	1,557 (6.9%)	
Hispanic	490 (5.5%)	1,641 (7.3%)	
NH American Indian/Alaska Native	27 (0.3%)	50 (0.2%)	
NH Hawaiian Native/Other Pacific Islander	13 (0.1%)	24 (0.1%)	
NH Multiracial	621 (6.9%)	1,447 (6.4%)	
**MCAT quartiles**			<0.001
1st (lowest)	758 (8.4%)	6,198 (27.5%)	
2nd	1,605 (17.9%)	7,505 (33.4%)	
3rd	1,762 (19.6%)	4,590 (20.4%)	
4th (highest)	4,851 (54.0%)	4,205 (18.7%)	
**Years to graduation**			<0.001
≤ 4 years	7,329 (81.7%)	20,207 (89.8%)	
> 4 years	1,647 (18.3%)	2,291 (10.2%)	
**Research experiences, quintiles Median, IQR**	3.00 (2.00-5.00)	3.00 (1.00-4.00)	<0.001
0-1	1,285 (14.3%)	6,075 (27.0%)	
2	1,757 (19.6%)	5,032 (22.4%)	
3-4	3,567 (39.7%)	7,446 (33.1%)	
>4	2,367 (26.4%)	3,945 (17.5%)	
**Publication count, quintiles Median, IQR**	5.00 (2.00-10.00)	3.00 (1.00-7.00)	<0.001
0-1	1,455 (16.2%)	6,805 (30.2%)	
2-4	2,470 (27.5%)	6,904 (30.7%)	
5-8	2,326 (25.9%)	4,939 (22.0%)	
>8	2,725 (30.4%)	3,850 (17.1%)	
**Publications per research experience (Median, IQR])**	1.60 (1.00-3.00)	1.25 (0.50-2.33)	<0.001

Abbreviations: NIH: National Institute of Health, NH: non-Hispanic, MCAT: Medical College Admission Test, IQR: inter-quartile range.

© 2022 Nguyen M et al. *JAMA Network Open.*

eTable 2. Characteristics of US MD Graduates Between 2018 to 2019 and 2019 to 2020 by MCAT Score and Time to Graduation

	Years to graduation		*p*-*value*	MCAT quartile				*p*-value
	≤ 4 years	> 4years		1st (Lowest)	2nd	3rd	4th (Highest)	
**Total: 31,474**	N=27,536	N=3,938		N=6,956	N=9,110	N=6,352	N=9,056	
**Sex**			0.024					<0.001
Male	14,340 (52.08%)	1,975 (50.15%)		2,873 (41.30%)	4,505 (49.45%)	3,446 (54.25%)	5,491 (60.63%)	
Female	13,196 (47.92%)	1,963 (49.85%)		4,083 (58.70%)	4,605 (50.55%)	2,906 (45.75%)	3,565 (39.37%)	
**Race/ethnicity**			<0.001					<0.001
NH White	16,908 (61.40%)	1,725 (43.80%)		3,184 (45.77%)	5,932 (65.12%)	4,047 (63.71%)	5,470 (60.40%)	
NH Asian	5,439 (19.75%)	990 (25.14%)		630 (9.06%)	1,581 (17.35%)	1,484 (23.36%)	2,734 (30.19%)	
NH Black/African American	1,605 (5.83%)	494 (12.54%)		1,394 (20.04%)	415 (4.56%)	162 (2.55%)	128 (1.41%)	
Hispanic	1,725 (6.26%)	406 (10.31%)		1,118 (16.07%)	566 (6.21%)	243 (3.83%)	204 (2.25%)	
NH American Indian/Alaska Native	64 (0.23%)	13 (0.33%)		39 (0.56%)	20 (0.22%)	10 (0.16%)	8 (0.09%)	
NH Hawaiian Native/Other Pacific Islander	29 (0.11%)	8 (0.20%)		13 (0.19%)	13 (0.14%)	3 (0.05%)	8 (0.09%)	
NH Multiracial	1,766 (6.41%)	302 (7.67%)		578 (8.31%)	583 (6.40%)	403 (6.34%)	504 (5.57%)	
**Sex-Race/ethnicity**			<0.001					<0.001
White male	9,177 (33.33%)	906 (23.01%)		1,364 (19.61%)	3,014 (33.08%)	2,264 (35.64%)	3,441 (38.00%)	
Asian male	2,683 (9.74%)	497 (12.62%)		230 (3.31%)	719 (7.89%)	718 (11.30%)	1,513 (16.71%)	
URiM male	2,480 (9.01%)	572 (14.53%)		1,279 (18.39%)	772 (8.47%)	464 (7.30%)	537 (5.93%)	
White female	7,731 (28.08%)	819 (20.80%)		1,820 (26.16%)	2,918 (32.03%)	1,783 (28.07%)	2,029 (22.41%)	
Asian female	2,756 (10.01%)	493 (12.52%)		400 (5.75%)	862 (9.46%)	766 (12.06%)	1,221 (13.48%)	
URiM female	2,709 (9.84%)	651 (16.53%)		1,863 (26.78%)	825 (9.06%)	357 (5.62%)	315 (3.48%)	

© 2022 Nguyen M et al. *JAMA Network Open.*

Abbreviations: NH: non-Hispanic, MCAT: Medical College Admission Test

© 2022 Nguyen M et al. *JAMA Network Open.*

eTable 3. Association of Sex and Race and Ethnicity With Publication Count Among 2018 to 2020 MD Graduates

	**Medical School NIH Research Ranking**					
	**Top 40**			**Not Top 40**		
	**Median (IQR)**	**Unadjusted IRR**	**Adjusted IRR: MCAT + Time to graduation**	**Median (IQR)**	**Unadjusted IRR**	**Adjusted IRR: MCAT + Time to graduation**
**Sex**						
Male	6.00 (2.00-10.00)	(Ref)	(Ref)	3.00 (1.00-7.00)	(Ref)	(Ref)
Female	5.00 (3.00-10.00)	**0.88 (0.86 - 0.89)**	**0.89 (0.87 - 0.90)**	3.00 (1.00-7.00)	**0.92 (0.91 - 0.93)**	**0.93 (0.92 - 0.95)**
**Race/ethnicity**						
White	5.00 (2.00-9.00)	(Ref)	(Ref)	3.00 (1.00-7.00)	(Ref)	(Ref)
Asian	7.00 (3.00-11.00)	**1.24 (1.22 - 1.27)**	**1.22 (1.20 - 1.24)**	4.00 (2.00-8.00)	**1.14 (1.12 - 1.16)**	**1.13 (1.12 - 1.15)**
Black/African American	4.00 (2.00-8.00)	**0.95 (0.92 - 0.98)**	1.01 (0.98 - 1.05)	2.00 (0.00-5.00)	**0.79 (0.77 - 0.81)**	**0.87 (0.84 - 0.89)**
Hispanic	4.00 (2.00-9.00)	**1.06 (1.02 - 1.1)**	**1.10 (1.06 - 1.14)**	2.00 (0.00-5.00)	**0.91 (0.88 - 0.93)**	**0.96 (0.94 - 0.99)**
American Indian/Alaska Native	5.00 (2.00-12.00)	0.87 (0.74 - 1.01)	0.91 (0.78 - 1.06)	3.00 (0.00-7.00)	**1.17 (1.03 - 1.33)**	**1.23 (1.09 - 1.39)**
Hawaiian Native/Other Pacific Islander	4.00 (2.00-13.00)	1.04 (0.85 - 1.28)	1.05 (0.85 - 1.29)	2.50 (0.50-5.00)	0.83 (0.67 - 1.02)	0.85 (0.69 - 1.05)
Multiracial	5.00 (3.00-10.00)	**1.11 (1.08 - 1.15)**	**1.13 (1.09 - 1.16)**	3.00 (1.00-6.00)	0.97 (0.95 - 1)	0.99 (0.97 - 1.02)

© 2022 Nguyen M et al. *JAMA Network Open.*
